# Development of the Fray-Farthing-Chen Cambridge Process: Towards the Sustainable Production of Titanium and Its Alloys

**DOI:** 10.1007/s11837-017-2664-4

**Published:** 2017-12-01

**Authors:** Di Hu, Aleksei Dolganov, Mingchan Ma, Biyash Bhattacharya, Matthew T. Bishop, George Z. Chen

**Affiliations:** 1 Department of Chemical and Environmental Engineering, Energy Engineering Research Group, Faculty of Science and Engineering, University of Nottingham Ningbo China, Ningbo, 315100 China; 2 International Doctoral Innovation Centre, University of Nottingham Ningbo China, Ningbo, 315100 China; 3 Department of Chemical and Environmental Engineering, Advanced Materials Research Group, Faculty of Engineering, University of Nottingham, Nottingham, NG7 2RD UK

## Abstract

The Kroll process has been employed for titanium extraction since the 1950s. It is a labour and energy intensive multi-step semi-batch process. The post-extraction processes for making the raw titanium into alloys and products are also excessive, including multiple remelting steps. Invented in the late 1990s, the Fray-Farthing-Chen (FFC) Cambridge process extracts titanium from solid oxides at lower energy consumption via electrochemical reduction in molten salts. Its ability to produce alloys and powders, while retaining the cathode shape also promises energy and material efficient manufacturing. Focusing on titanium and its alloys, this article reviews the recent development of the FFC-Cambridge process in two aspects, (1) resource and process sustainability and (2) advanced post-extraction processing.

**Electronic supplementary material:**

The online version of this article (10.1007/s11837-017-2664-4) contains supplementary material, which is available to authorized users.

## Introduction

Titanium and its alloys exhibit excellent properties; including high specific strength, biocompatibility, and resistance to extreme conditions.^1^ However, their high costs have placed them in niche markets such as aerospace, medical implants, and offshore applications.^2^ Affordable production of titanium and its alloys has been pursued since the Kroll process was first commercialised in the early 1950s.^3^ The throughput from the Kroll Process for titanium extraction has been increased to some extent compared to that of the formerly used Hunter Process,^4^ and several innovations have been applied to increase the efficiency.^5^ Nonetheless, this process is still an inherently labor- and energy-intensive (energy consumption: ca. 50 kWh/kg Ti^6^), environmentally unfriendly (> 2 kg CO_2_ per kg Ti) and a semi-batch process. Thus, there have been continuous research and industrial efforts to improve or replace the Kroll process^7^–^22^ as summarised in the supplementary Table S1.

In addition to extraction, the fabrication of titanium into its alloys and final products has many obstacles to overcome, owing to titanium and its alloys having high affinity to oxygen and poor machinability.^23^ For example, the cost of post-extraction processes (i.e., from arc melting to fabrication) accounts for 62% of the total cost for producing a 2.54 cm thick titanium alloy plate as illustrated in supplementary Fig. S1.^8^ Due to the relatively low density of titanium, some alloying elements tend to segregate and multi-step remelting is necessary to achieve full homogenisation of the final alloys at high costs. Furthermore, the fabrication of titanium alloys in complex shapes increases both the waste and cost and calls for creative manufacturing techniques such as additive manufacturing,^24^–^26^ near-net-shape casting,^27^ spark plasma sintering (SPS),^28^ and metal injection moulding.^29^ Most of these advanced techniques are based on powder metallurgy,^30^^,^ 31 and powder production requires sophisticated pulverisation and spheroidisation processes, which in turn adds extra costs to the final products.

For a sustainable and affordable titanium industry, process evolution has become necessary, which may come from two directions:^32^ (1) resource and process sustainable extraction of titanium and (2) advanced manufacturing of titanium alloys and their final products. This paper provides an overview of one of the promising extractive electrometallurgy techniques, i.e., the Fray-Farthing-Chen (FFC) Cambridge process, focusing on the aspects related to a sustainable titanium industry.

## Concept of the FFC-Cambridge Process

The FFC-Cambridge process was first established on the electro-reduction of TiO_2_ to pure titanium in molten calcium chloride (CaCl_2_),^15^ and now it has been applied to reduce a variety of metal compounds, particularly oxides, to their respective metals, alloys and intermetallic compounds.^6^^,^ 33–48 In the process, the preformed metal compound (e.g., pellet of TiO_2_) is attached on a cathode which is then electrolysed against a suitable anode under a cell voltage that is high enough to ionise the oxygen in the metal compound without decomposing the electrolyte (e.g., molten CaCl_2_). The FFC-Cambridge process can be represented by the following reactions where M represents a metal.^8^

Overall reactions:

R1 $$ {\text{MO}}_{x} ({\text{s}}) = {\text{M}}({\text{s}}) + x /2{\text{O}}_{2} ({\text{g}})\;\left( {{\text{using}}\;{\text{an}}\;{\text{inert}}\;{\text{anode}}} \right) $$

R2 $$ n{\text{MO}}_{x} ({\text{s}}) + x{\text{C}}({\text{s}}) = n{\text{M}}({\text{s}}) + x{\text{CO}}_{n} ({\text{g}})\;\left( {{\text{using}}\;{\text{a}}\;{\text{carbon}}\;{\text{anode}},\;n = 1\;{\text{or}}\;2} \right) $$

Cathode reaction:

R3 $$ {\text{MO}}_{x} ({\text{s}}) + 2x{\text{e}}^{ - } = {\text{M}}({\text{s}}) + x{\text{O}}^{2 - } $$

Anode reactions:

R4 $$ x{\text{O}}^{2 - } = x /2{\text{O}}_{2} ({\text{g}}) + 2x{\text{e}}^{ - } \;\left( {{\text{using}}\;{\text{an}}\;{\text{inert}}\;{\text{anode}}} \right) $$

R5 $$ n{\text{O}}^{2 - } + {\text{C}}({\text{s}}) = {\text{CO}}_{n} ({\text{g}}) + 2n{\text{e}}^{ - } \;\left( {{\text{using}}\;{\text{a}}\;{\text{carbon}}\;{\text{anode}},\;n = 1\;{\text{or}}\;2} \right) $$

**Fig. 1:**
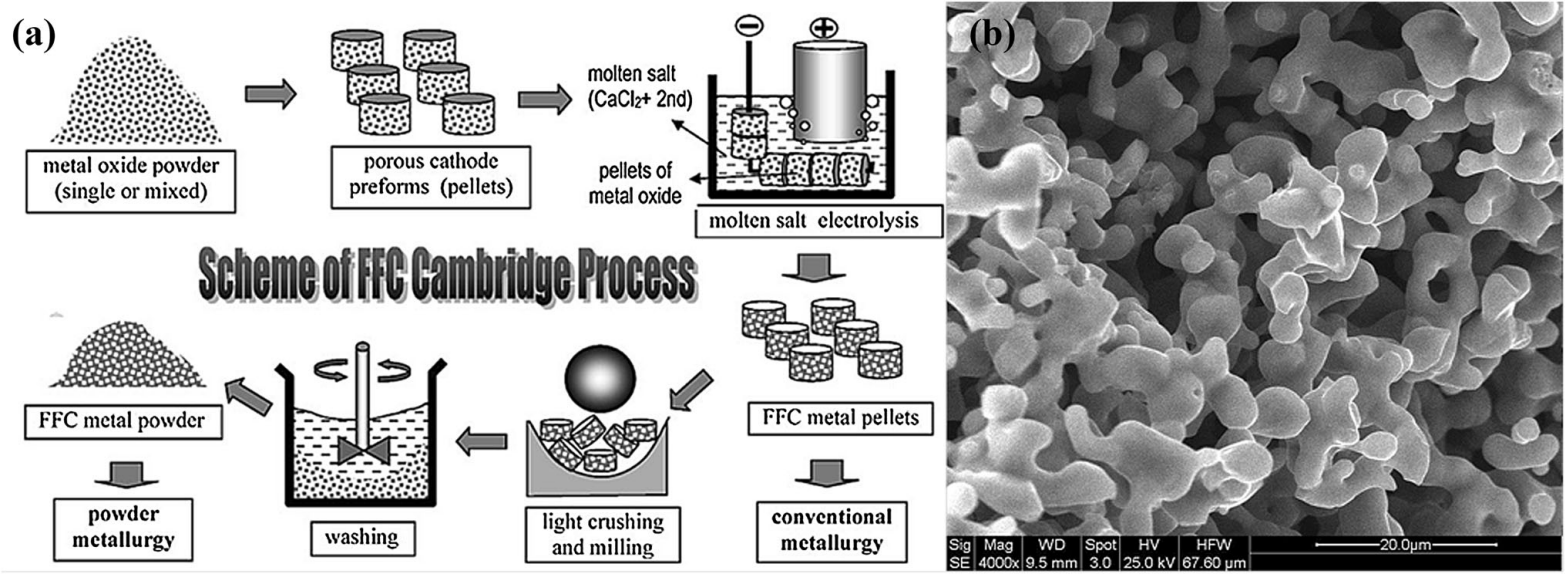
(a) An illustration of the FFC-Cambridge process for the electrochemical reduction of solid metal oxide to solid metal in molten salt. (b) The microstructure of a titanium sample produced by the same process in the authors’ laboratory. (a) Reprinted from^49^ with permission

Figure 1a illustrates schematically the FFC-Cambridge process^49^ and Fig. 1b shows the typical porous and interconnected microstructure of the as produced FFC titanium. This morphology results from the reconstructive phase transformations during the complex kinetic pathway for deoxidation of TiO_2_, and the in situ sintering of the formed titanium fine particles,^50^^,^ 51 which can be pulverised to powders for further treatments or applications.^52^

## Towards Higher Efficiency for Titanium Extraction

During electrolysis in molten CaCl_2_, once TiO_2_ is partially reduced to TiO_*x*_ (1 < *x* ≤ 2), the discharged oxygen anions (O^2−^) and nearby calcium cations (Ca^2+^) tend to combine with it, either chemically or electrochemically, to form perovskites (Ca_*σ*_TiO_*x*_, *x*/*σ* ≥ 2).^42^^,^ 50 This intermediate step is named as in situ perovskitisation.^42^ The suggested pathway is given in supplementary materials (SM).^50^

The problem of in situ perovskitisation is that it reduces the porosity and slows O^2−^ transport through the pores between the oxide particles.^42^^,^ 53 It is believed that CaO plays an important role in this process.^54^^,^ 55 A low O^2−^ concentration may force the oxidation of chloride ions (Cl^−^) to chlorine gas on the anode during initial electrolysis stages.^50^^,^ 54 Thus, an appropriate initial CaO concentration could mitigate the transport limitation of O^2−^ ions and increase the current density.^54^ Moreover, an intrinsic barrier to the electro-reduction of TiO_2_ to titanium persists, i.e., the oxide-to-metal molar volume ratio which is known as the Pilling-Bedworth ratio (PBR) (cf. SM).^56^ This ratio is commonly used for analysis of metal oxidation in hot air, whilst it should also help understand the reversed process, i.e., electro-reduction of metal oxides.^57^

Since these kinetic barriers persist in the FFC-Cambridge process, despite an acceptable level of energy consumption (ca. 33 kWh/kg Ti versus ca. 50 kWh/kg Ti for the Kroll process),^57^ the current efficiency is still low (e.g., 15% to achieve ≤ 3000 ppm oxygen in Ti)^42^^,^ 54 ^,^ 57 when compared with that for chromium (Cr) extraction (e.g., > 70% @ < 2000 ppm oxygen in Cr)^49^ and zirconium (Zr) extraction (e.g., 45% @ 1800 ppm oxygen in Zr).^58^

To cope with these issues, some improvements in the FFC-Cambridge process have been made,^42^^,^ 54 ^,^ 57 as elaborated below.

1. As previously described, a sufficient amount of O^2−^ (which can be in the form of CaO) is required during initial electrolysis stages for electro-reduction of TiO_2_.^54^ Also, the releasing rate of O^2−^ during different electrolysis stages must be carefully controlled to avoid local CaO saturation at the cathode, which would slow or even stall the electrolysis.^54^^,^ 55 Therefore, a combination electrolyte, CaCl_2_ + 2 mol.% CaO, has been utilised.^54^^,^ 59 It was reported that, for 16 h of electrolysis, the titanium samples with 2000 ppm to 5000 ppm oxygen were produced with a current efficiency ranging from 10% to 40%.^54^
2. In order to avoid in situ perovskitisation, ex situ perovskitisation has been introduced.^42^ This process is carried out by pre-mixing and sintering TiO_2_ and CaO/calcium hydroxide (Ca(OH)_2_) to form the perovskite precursor (e.g., calcium titanate (CaTiO_3_)). It was found that direct electro-reduction of the formed CaTiO_3_ (10 h of electrolysis, 2100 ppm oxygen in titanium)^42^ was significantly faster than that of the TiO_2_ precursor (16 h of electrolysis, 2400 ppm oxygen in titanium).^54^ However, this process releases CaO to the melt, which requires purification steps for industrial applications.
3. Despite the intrinsic barrier of PBR, titanium can dissolve oxygen to form solid solutions. Although the oxygen diffusion rate in titanium at a given temperature is fixed, the removal of dissolved oxygen can be accelerated by increasing the porosity of the TiO_2_ precursor, which will enlarge the titanium/molten CaCl_2_ interface.^57^ The increased porosity can also mitigate the local saturation of O^2−^ ions in the pores of TiO_2_ and its subsequent reactions to form CaO and perovskites (cf. supplementary RS1 to RS5). Ammonium bicarbonate (NH_4_HCO_3_) was utilised as a cheap and recyclable fugitive pore forming agent to fabricate high-porosity TiO_2_ precursors.^57^ Nevertheless, the increased porosity and subsequent high surface area of the electrolytic product can increase the oxygen content during washing to remove the solidified salt. A two-step procedure was therefore introduced, including a high voltage reduction step (e.g., electrolysis at 3.2 V for 3 h at 850°C) and a low voltage in situ sintering step (e.g., electrolysis at 2.6 V for 3 h at 850°C).^57^ With this improvement, the energy consumption and current efficiency for extracting titanium with 1900 ppm oxygen were 21.5 kWh/kg and 32.3%, respectively.^57^

These improvements are summarised in supplementary Table S2. A remaining challenge to the FFC-Cambridge process for titanium extraction is the lower current efficiency. This can be ascribed partly to electronic conduction in the molten salt, due to dissolved calcium metal in CaCl_2_ at less than unit activity^54^^,^ 55 ^,^ 60–62 and the presence of redox-active impurities,^63^ although the latter can be largely removed by pre-electrolysis. Further understanding is still required to overcome these obstacles and it is anticipated that by careful control of the electrolysis conditions, the energy consumption and current efficiency should reach 12.5 kWh/kg and 50% to achieve 2000 ppm oxygen.^60^

## Towards Resources Sustainability

### A More Sustainable Feedstock

For titanium production, the FFC-Cambridge process commonly uses pigment grade TiO_2_ as the feedstock, which is safer to handle and transport than TiCl_4_ used by the Kroll process. Although the price of pigment grade TiO_2_ is typically double of that of TiCl_4_, it only needs 1.66 kg of TiO_2_ to produce 1 kg of titanium whereas 4 kg of TiCl_4_ is required for the same yield. However, pigment grade TiO_2_ is produced by either the chloride or sulphate process (cf. supplementary RS8 to RS9 and RS10 to RS12), and both cause environmental concerns.^64^

In particular, for TiCl_4_ production, the carbochlorination process uses hazardous chemicals and substantial quantities of energy, and emits carbon oxides. It also requires high-grade natural rutile which is rapidly depleting.^64^ Consequently, the exploitation of a more sustainable and low-cost resource for the FFC-Cambridge process has been deployed.

In 2006, it was demonstrated that titanium, with < 3000 ppm oxygen and low metallic impurities, can be extracted directly from titania dust (collected from the floor near the rotary kiln in a titania processing plant) and metatitanic acid (in solid state), via the FFC-Cambridge process.^43^ Utilisation of these low-cost feedstocks can reduce the environmental impact, and are worth further research and development.

Moreover, titanium-rich slag,^43^^,^ 65 synthetic and natural ilmenite ore, containing Fe, Si, Mg, Ca, Mn, and Al,^45^^,^ 46 ^,^ 66 were also successfully reduced to ferrotitanium alloys. The supplementary Fig. S3a and S3b show the microstructures of the ground natural ilmenite feedstock before and after electro-reduction, respectively.^66^

More recently, low-cost and novel titanium alloys were produced directly from either synthetic rutile (i.e., rutile produced from ilmenite, with a transition metal element concentration of 3.7% and aluminium content below 1%),^21^^,^ 67 or naturally occurring rutile ore (beach sand)^20^ via the FFC-Cambridge process (see supplementary Fig. S3c and S3d).^67^ The obtained titanium alloy powder was spheroidised, and fabricated into a billet via hot isostatic pressing (HIP) and subjected to monotonic tensile testing.^20^ The test result revealed that the ultimate tensile strength of this material is close to that of Ti-6Al-4V.^20^

In summary, the FFC-Cambridge process can use various cheap and recycled feedstocks to produce titanium and specific alloys, making it a more resource sustainable and environmentally friendly process. These alternative and low-cost feedstocks currently find little or no applications, but feeding them into the FFC-Cambridge process surely increases their values.

### Regeneration and Cathodic Protection of Titanium and Its Alloys

**Fig. 2:**
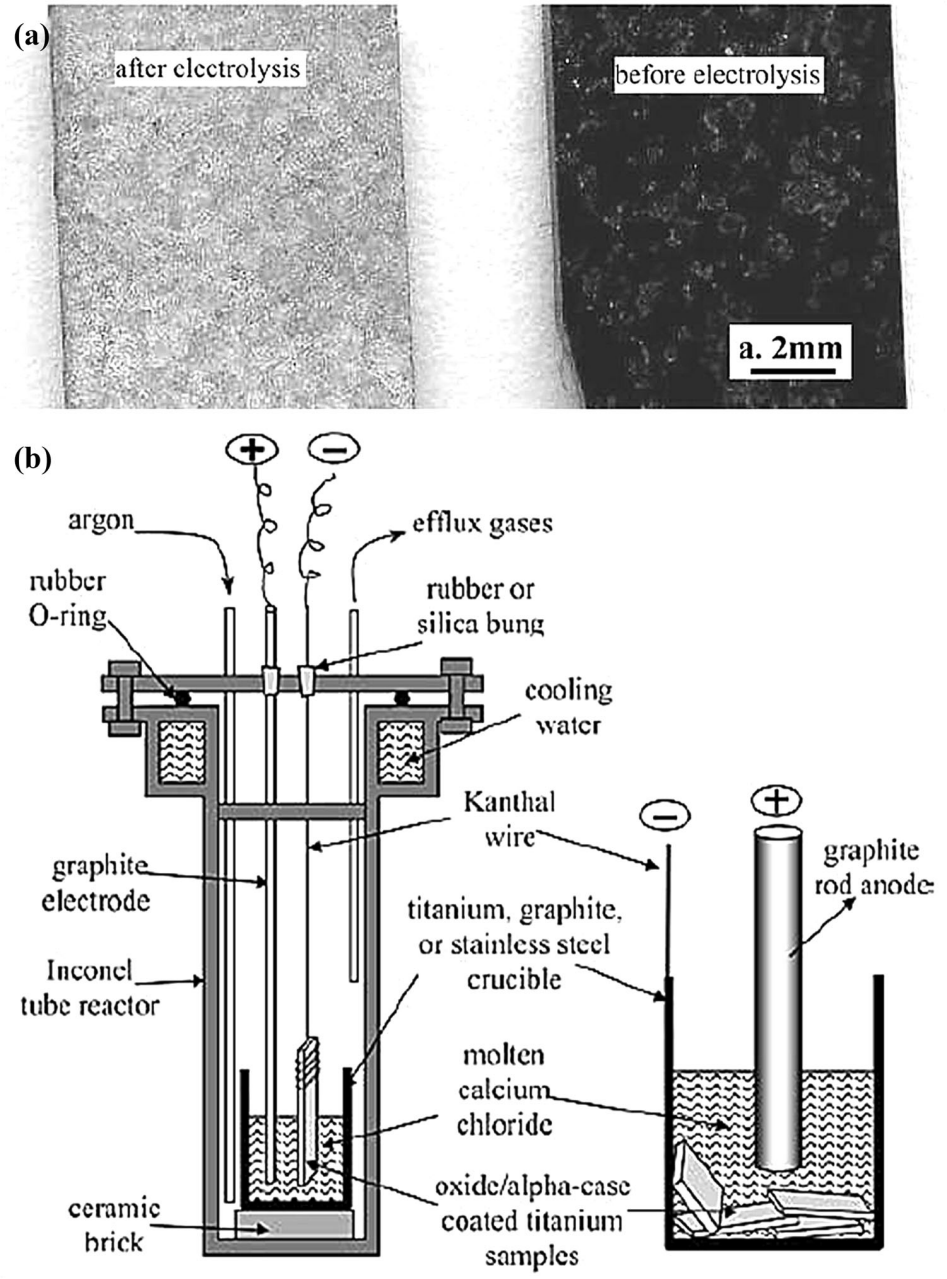
(a) Optical scanning image of an alpha-case covered Ti-6Al-4V foil (right) before and (left) after electro-reduction in molten CaCl_2_ at 3.0 V and 950°C for 1 h. (b) Reactor and electrolytic cell for removal of the alpha-case on titanium and alloys in molten CaCl_2_, showing (left) a sample suspended in molten salt and (right) more samples placed at the bottom of the crucible. Reprinted from^69^ with permission

When subjected to hot processing in air, titanium and its alloys can form a layer of solid oxygen solution, i.e., the alpha-case underneath the surface oxide scale. The alpha-case is brittle and can severely deteriorate the performances of titanium components.^68^ Current methods for removing this alpha-case include pickling in acid, and grinding, which inevitably change the original dimensions of the component, and add costs and environmental burdens to titanium manufacturing. It was demonstrated that under almost identical conditions for electro-reduction of TiO_2_ (cf. Figs. 1a and 2b) but replacing TiO_2_ by the alpha-case covered titanium or its alloy samples, the alpha-case can be effectively converted to a low-oxygen metal phase by the FFC-Cambridge process as shown in Fig. 2a.^69^ This work implies an alternative, simpler and more material efficient way to either regenerate spent titanium components without affecting their dimensions, or recycle titanium scraps.^69^

Recently, this cathodic refining concept was adopted for cathodic protection of titanium alloys from being oxidised in hot air using molten salt fluxes as the electrolyte.^70^ In this work, the titanium alloy was the solid or liquid cathode, coupled with an oxygen-evolving anode of iridium (Ir) which is inert under these working conditions.^70^ Further development of this method has led to a novel idea of laser welding titanium alloy in air without using a protective gas.^71^

## Towards the Processes Sustainability

### A Carbon-Free Titanium Extraction Process

The use of carbon based anodes in the FFC-Cambridge process leads to evolution of carbon oxides (cf. R5) and carbon dioxides (CO_2_) can react with O^2−^ ions in the molten salt to form carbonate ions (CO_3_^2−^) which can then transfer to, and be reduced to carbon at the cathode.^22^^,^ 54 ^,^ 72 These parasitic reactions lower the current efficiency and cause contamination to the cathodic products via, e.g., carbide formation.^22^ When evolving gases, the carbon anode may also release carbon debris that float on the molten salt surface and potentially short circuit the cell, further impairing the current efficiency.^59^ Thus, by replacing carbon with an inert material, pure oxygen can evolve as the only anodic off-gas (cf. R4), and current and energy efficiency and product quality can all improve.

Among the various metallic and ceramic materials tested, including cermets, doped tin oxide (SnO_2_)^73^^,^ 74 and the solid solution of CaTiO_3_ and calcium ruthenate (CaRuO_3_) (i.e., CaTi_*x*_Ru_1−*x*_O_3_)^75^^,^ 76 are reviewed here as the candidates of a proper inert anode material for the FFC-Cambridge process. The antimony oxide (Sb_2_O_3_) (electrical conductivity enhancer) and copper oxide (CuO) (densification enhancer) doped SnO_2_ 77 was initially tested for making an inert anode in cryolite-alumina melts.^78^ Using the same anode, successful reduction of tantalum pentoxide (Ta_2_O_5_) to tantalum metal via the FFC-Cambridge process was achieved, although tin contamination was observed in the cathodic product.^74^ The use of the doped SnO_2_ anode can result in improved current efficiency and a cleaner electrolyte when compared to that of a carbon anode.^74^ However, an insulating layer of calcium stannate (CaSnO_3_) formed on the anode surface after 24 h electrolysis, which ultimately terminated the operation.^22^^,^ 73 ^,^ 74

CaRuO_3_ was tested as the inert anode material to evolve pure oxygen during electro-reduction of TiO_2_ and proven highly stable in chloride melts (see supplementary Fig. S4a and S4b).^59^^,^ 75 ^,^ 76 However, CaRuO_3_ alone is too expensive to use, whilst CaTiO_3_ is too resistive. Therefore, the cheaper CaTiO_3_ and the highly conductive CaRuO_3_ were utilised to form the solid solution of CaTiO_3_-CaRuO_3_, i.e., CaTi_*x*_Ru_1−*x*_O_3_, which was then made into the inert anode for titanium and titanium-nickel (Ti-Ni) alloy production.^41^^,^ 76 The CaTi_*x*_Ru_1−*x*_O_3_ inert anodes exhibited no noticeable erosion/corrosion or formation of an insulating layer after use (see supplementary Fig. S4c and S4d). The corrosion rate of this inert anode was calculated as only 0.0015 g/cm^2^/h in molten CaCl_2_ containing 1 wt.% CaO.^76^

By combining an inert anode with the optimised processing conditions, within a timeframe of 14-16 h, the energy consumption and current efficiency for titanium extraction via the FFC-Cambridge process can be ca. 17 kWh/kg Ti and ca. 40%, respectively.^59^

Additionally, the Solid Oxide Membrane (SOM) process^16^ has also shown the ability to eliminate the carbon related issues for titanium alloy production^17^ (cf. SM).

### An Affordable Alloying Process

The FFC-Cambridge process can be fed with mixed metal oxides at a predefined ratio to produce an alloy in one step. Its simplicity over the conventional process for Ti-Ni fabrication is exemplified in supplementary Fig. S5.^6^

Due to its solid state reactions, the FFC-Cambridge process can make alloys that are either impossible, or challenging to make by conventional processes, such as those with alloying elements with vastly mismatching densities, melting points and vapour pressures.^60^^,^ 79 The titanium-tungsten (Ti-W) alloy can make effective implants because of their low cytotoxicity, superior wear resistance and strength, and relatively low elastic modulus.^34^^,^ 35 ^,^ 80 ^,^ 81 However, fabrication of Ti-W alloys is not viable by melt processing, as the melting point of tungsten (3422°C) is higher than the boiling point of titanium (3287°C). Additionally, tungsten has a huge difference in density to titanium (19,250 kg/m^3^ versus 4505 kg/m^3^) which can cause segregation of the alloying elements during melting and the following liquid processing. Although powder metallurgy has been used,^80^–^82^ in order to overcome the extremely sluggish diffusion kinetics of tungsten during consolidation, fine titanium and tungsten particles have to be used which will inevitably increase the oxygen content. To address these issues, the FFC-Cambridge process has therefore been successfully employed to fabricate Ti-W alloys in one step.^34^^,^ 35 ^,^ 81

Since its initial conception, the FFC-Cambridge process has been used to fabricate numerous titanium alloys, such as Ti-6Al-4V,^36^ Ni-35Ti-15Hf,^37^ Ti-10V-2Fe-3Al,^20^^,^ 38 Ti-W^34^^,^ 35 ^,^ 81 Ti-Ni,^6^^,^ 39–41 Ti-Fe,^42^–^46^ and Ti-Mo.^20^^,^ 47 It was also noted that the α- and β-phases in the Ti-Zr alloys could be easily tuned by controlling the electrolysis duration, which adjusts the oxygen content in the Ti-Zr alloys.^48^

Most recently, the high-entropy alloys (e.g., TiNbTaZr and TiNbTaZrHf) have been fabricated using the FFC-Cambridge process, which further demonstrates its capabilities for alloy making.^83^

## Incorporation with the Advanced Manufacturing Concepts

One of the key areas of recent development within the titanium industry is powder metallurgy (e.g., 3D printing^25^^,^ 26 and near-net-shape manufacturing^31^). Since titanium and its alloys produced from the FFC-Cambridge process are typically in a porous structure (see Fig. 1b), they can be pulverised and used as the feedstock for powder metallurgy. This potential was investigated by Metalysis™ (Rotherham, UK) through direct grinding of the electrolytic titanium, hydriding-grinding-dehydriding, and fusion and gas atomisation.^52^

**Fig. 3:**
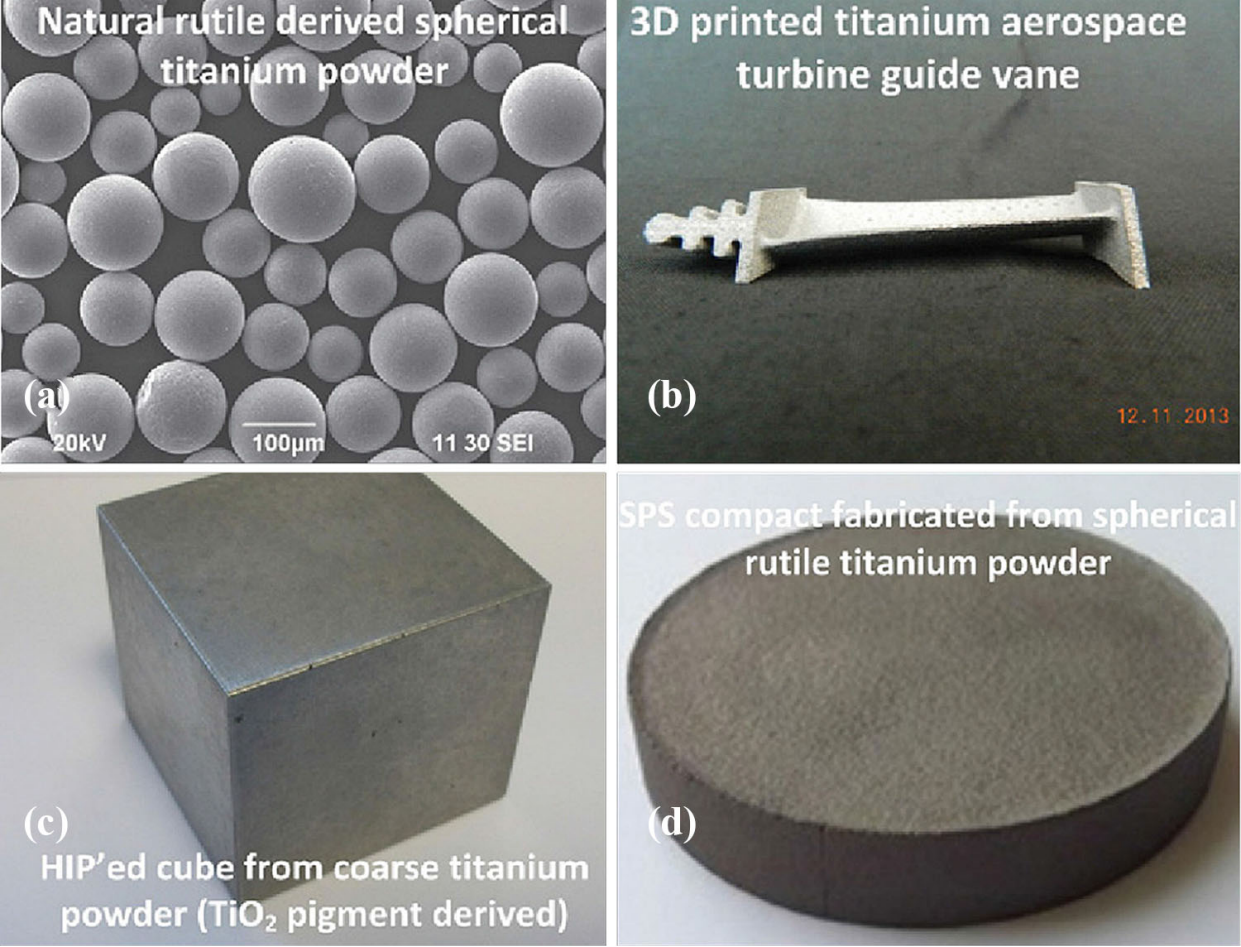
(a) SEM image of plasma spheroidised titanium powder, and photographs of (b) a 3D printed aerospace turbine guide vane using spherical titanium powders, (c) HIP’ed cube from coarse titanium powder extracted from pigment grade TiO_2_, and (d) SPS compact from spherical titanium powder extracted from rutile. Reproduced from^20^ with permission

Recently, Metalysis™ also attempted direct electro-reduction of natural rutile powders to titanium powders.^20^ Following plasma spheroidisation (see Fig. 3a) and 3D printing of the electrolytic powders, affordable titanium components were made.^20^^,^ 21 Figure 3b shows a 3D printed aerospace turbine guide vane using Metalysis™ spherical titanium powders.^20^^,^ 21 The workability of the electrolytic titanium powders was also evaluated using different shaping techniques, e.g., HIP (Fig. 3c), and SPS (Fig. 3d).^20^^,^ 28

**Fig. 4:**
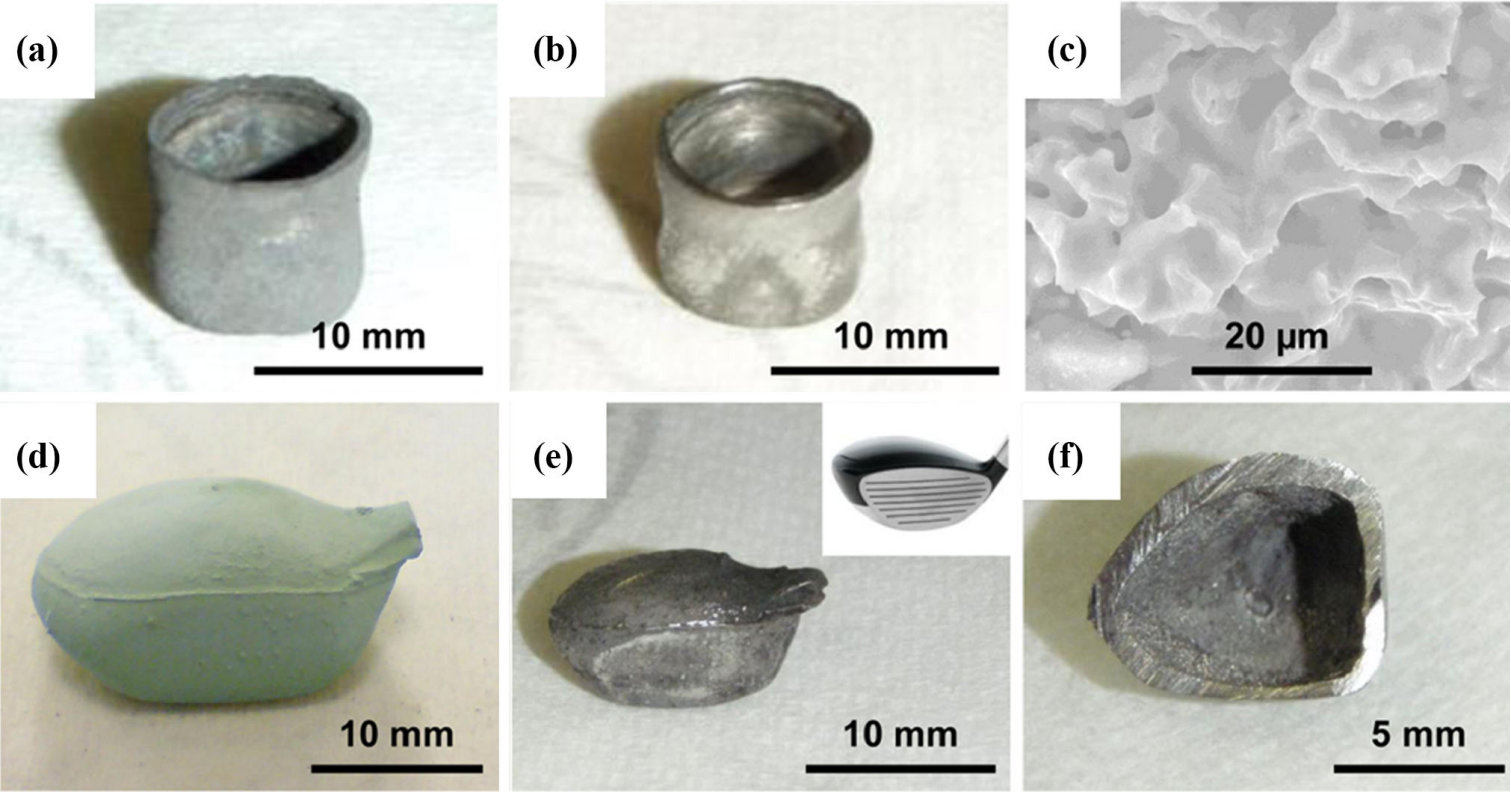
Photographs of electrolytic Ti-6Al-4V components. (a) A cylindrical cup before and (b) after polishing, and (c) SEM image of its well consolidated interior structure. (d) A miniature hollow golf club head made of metal oxide mixture before and (e) after electro-reduction. The insert in (e) is the photo of a real golf club head for shape comparison. (f) Cross section of electrolytically produced miniature hollow golf club head. Reproduced from^36^ with permission

Another feature of the FFC-Cambridge process is that it proceeds in the solid state, and the electrolytic products retain the shape closely to the original shape of the oxide precursors, although shrinkage would occur.^36^^,^ 58 ^,^ 84 By taking advantage of this unique ability, different shapes of Ti-6Al-4V components (such as hollow spheres, hollow miniature golf club heads, and cylindrical cups) were produced from their slip-cast oxide precursors.^36^ Figure 4 displays the photographs of different near-net-shape products from the FFC-Cambridge process.^36^

The versatility of the FFC-Cambridge process for near-net-shape production has been further demonstrated by fabricating hierarchically structured titanium foams for tissue scaffold applications,^84^ and Zr and Zr-2.5Nb tubes for nuclear reactor application.^58^

## Conclusion

Understanding of the mechanisms and kinetic barriers of the FFC-Cambridge process has progressed steadily in recent years, leading to the production of titanium with < 2000 ppm oxygen at 32.3% in currently efficiency and 21.5 kWh/kg in energy consumption. The process has the capability to combine different metallurgical steps, including metal extraction, alloying, and shaping, into one step. This has been shown to dramatically improve almost every aspect for sustainable and affordable production of titanium and its alloys.

Regarding resource sustainability, the process can handle different lower cost feedstocks, recycle titanium wastes and offer cathodic protection of titanium artefacts when subjecting these materials to hot processing in air. Process sustainability may be achieved via replacing the carbon anode by an inert anode. Different materials have been evaluated, showing CaTiO_3_-CaRuO_3_ to be most promising in terms of service life and cost. In addition, using the inert anode has also improved the current efficiency and product purity, improving the process sustainability.

Products from the process can be powdery or of a similar shape as the oxide precursor. Subjected to spheroidisation, the powder can be fed into 3D printing, SPS, and HIP. The shape retention ability has enabled direct conversion of metal oxide precursors with complex shapes into final titanium alloy components, i.e., near-net-shape production.

## Outlook

It should be pointed out that, like many discoveries and inventions, the laboratory research that led to the FFC-Cambridge process has been based on, and benefited from many past research and industrial achievements and failures.^85^–^93^ Now, the FFC-Cambridge process has been in industrial trial for over 16 years,^94^^,^ 95 and the developments are steady and promising toward a bright future (cf. Metalysis™ and GLABAT™ in SM).

## Electronic supplementary material

Below is the link to the electronic supplementary material.

Supplementary material 1 (PDF 562 kb)
