# Supplementary material for: Development of the Fray-Farthing-Chen Cambridge Process: Towards the Sustainable Production of Titanium and Its Alloys
Source: JOM (1989). 2017 Dec 1;70(2):129–37. doi: 10.1007/s11837-017-2664-4 (PMC6956875; doi:10.1007/s11837-017-2664-4)
Supplement: Supplementary file 1 — Supplementary material 1 (PDF 562 kb) [file 11837_2017_2664_MOESM1_ESM.pdf]

Supplementary Material: Development of the Fray-Farthing-Chen Cambridge Process: Towards the Sustainable Production of Titanium and Its Alloys

DI HU[1, 2, 4], ALEKSEI DOLGANOV[1], MINGCHAN MA[1], BIYASH BHATTACHARYA[1], MATTHEW T. BISHOP[1, 2], and GEORGE Z. CHEN[1, 2, 3, 4]

1.--Department of Chemical and Environmental Engineering, and Energy Engineering Research Group, Faculty of Science and Engineering, The University of Nottingham Ningbo China, Ningbo 315100, China.

2.--International Doctoral Innovation Centre, The University of Nottingham Ningbo China, Ningbo 315100, China.

3.--Department of Chemical and Environmental Engineering, and Advanced Materials Research Group, Faculty of Engineering, University of Nottingham, Nottingham NG7 2RD, UK.

4.--e-mails: di.hu@nottingham.edu.cn (Di Hu); george.chen@nottingham.ac.uk (George Z. Chen)

Table S1. Novel extraction technologies for titanium production [1-3]

Name	Non-electrolytic process	Products
The innovations in the Kroll Process itself [4]	(i) Fluidized bed chlorination; (ii) increased batch size; (iii) combined process technology; (vi) sponge handling and evaluation techniques; (v) development of energy-efficient cells	Sponge
Preform Reduction Process (PRP) [5]	Reduction of TiO_2 by Ca vapour	Powder compact
Mechanochemical Process [6]	Mechanical energy activated chemical reactions and structural changes. (i) Ambient temperature CaH_2 reduction of TiO_2; (ii) ambient temperature Mg reduction of $TiCl_4$.	Powder
Armstrong Process [7]	Liquid Na reduction of $TiCl_4$ vapor	Powder
**Name**	**Electrolytic process**	**Products**
Electronically Mediated Reaction (EMR) Process [8]	Electrolytic cell between TiO_2 and liquid Ca alloy reduces TiO_2	Highly porous Ti powder compact
Materials and Electrochemical Research (MER) Process [9]	Dissolution of titanium cations based composite anode, transport through mixed halide electrolyte and deposition on cathode	Powder, flake or solid slab
Ono-Suzuki (OS) Process [10]	Electrochemical-calciothermic reduction of TiO_2	Powder / sponge
Fray-Farthing-Chen-Cambridge Process (FFC-Cambridge Process) [11-12]	Electrolytic reduction of TiO_2 electrode in molten salt	Powder block
Solid Oxide Membrane (SOM) Process [13-14]*	Electrolytic reduction of dissolved Ti ions in a fluoride flux or solid TiO_2 in a molten salt. Using oxygen ion conducting yttrium stabilised zirconia (YSZ) membrane to separate the anode from the molten electrolyte.	Deposited Ti / powder block
Molten Oxide Electrolysis (MOE) Process [15-16]	Direct electrolysis of molten TiO_2	Liquid Ti

* For deoxidation of TiO_2, the cathodic reactions of the SOM process is identical to that of the FFC-Cambridge process, whereas its anodic reactions involve the removal of oxygen ions from the electrolyte via an oxide-ion-conducting solid electrolyte which separates the anodic reaction area from the molten electrolyte [13-14]. The carbon related side reactions are therefore confined within the tube of YSZ membrane, eliminating the carbon issues associated with the FFC-Cambridge process as described in the main text. However, because of the utilisation of an oxide-ion-conducting solid electrolyte, a high over-potential is therefore necessary, which results in a relatively higher cell voltage when compared with that of the FFC-Cambridge process (e.g. 3.8 V vs. 3.2 V for titanium production) [11, 14].

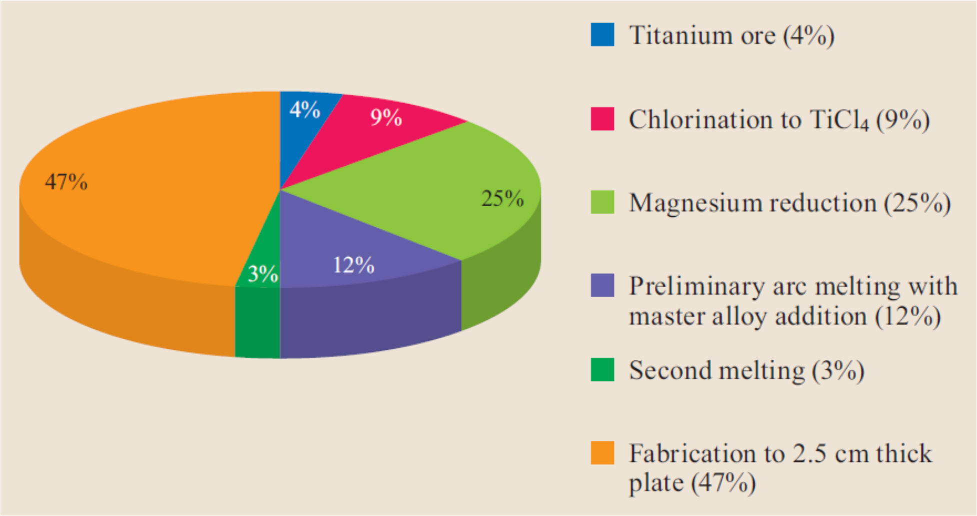

Fig. S1. Relative cost factors for conventional mill processing of a 2.54 cm thick Ti alloy plate (reprinted from [2] with permission).

The pathway of in situ perovskitisation [17]:

$5TiO_2(s) + Ca^{2+} + 2e^- = Ti_4O_7(s) + CaTiO_3(s)$	RS1
$4Ti_4O_7(s) + Ca^{2+} + 2e^- = 5Ti_3O_5(s) + CaTiO_3(s)$	RS2
$3Ti_3O_5(s) + Ca^{2+} + 2e^- = 4Ti_2O_3(s) + CaTiO_3(s)$	RS3
$2Ti_2O_3(s) + Ca^{2+} + 2e^- = 3TiO(s) + CaTiO_3(s)$	RS4
$CaTiO_3(s) + TiO = CaTi_2O_4(s)$	RS5
$CaTi_2O_4(s) + 2e^- = 2TiO(s) + Ca^{2+} + 2O^{2-}$	RS6
$TiO(s) + 2(1-\delta)e^- = Ti_{[O]\delta} + (1- \delta)O^{2-}$	RS7

The electrochemical reduction of TiO_2 involves the consumption of CaO during the early stages, such as the incorporation of calcium ions into the oxide precursor (cf. **RS1** to **RS4**) meanwhile discharging oxygen ions at the anode, and production of CaO during the late stages, such as decomposition of calcium titanite ($CaTi_2O_4$) to titanium monoxide (TiO) and CaO (cf. **RS6**).

Pilling-Bedworth Ratio (PBR)

$PBR = V_o/V_m$ E1

$V_o = M_o/n\rho_o$ E2

$V_m = M_m/\rho_m$ E3

Where the subscripts o and m represent the oxide and metal, respectively, V the molar volume, M the molar mass, ρ the density, and n the number of metal atoms in the oxide formula, e.g. n = 1 for TiO_2, n = 2 for aluminium oxide (Al_2O_3).

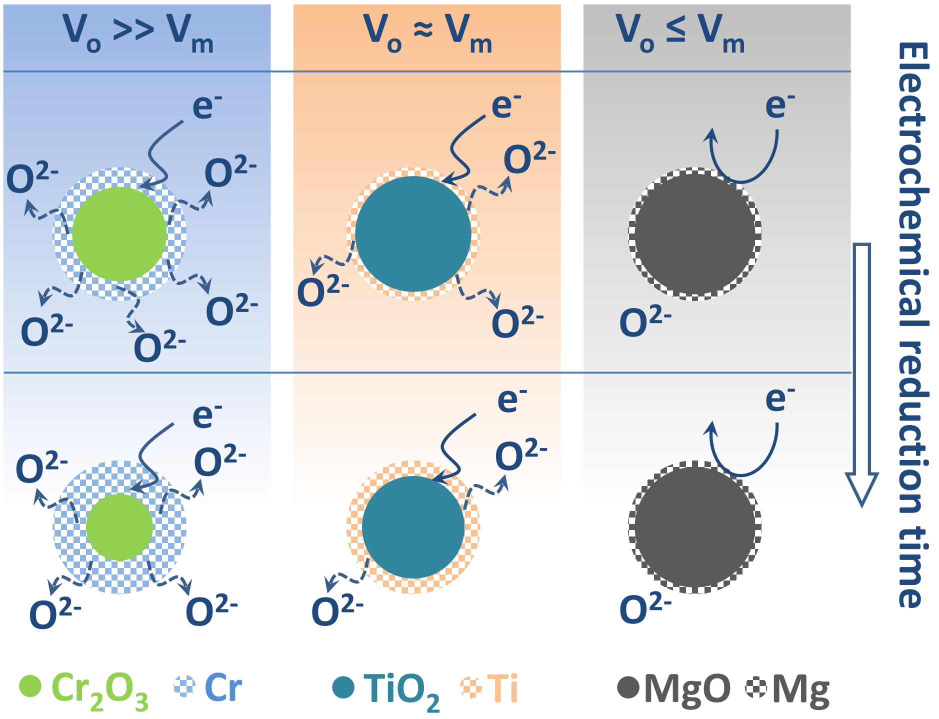

Fig. S2. An illustration shows the influence of PBR on electrochemical reduction of Cr_2O_3, TiO_2, and MgO, respectively (reproduced from [18] with permission).

As illustrated in **Fig. S2**, when the PBR is greater than unity, i.e. $V_o > V_m$, a porous metal layer can be generated from the removal of oxygen. This porous layer is essential for the electrochemical reduction process as it could allow molten salt to access the underlying metal oxide. Thus, the reduction-generated oxygen ions (O^{2-}) could be removed through the electrolyte in the pores of the metal layer, whist the solid state reduction of metal oxide to its metal could proceed (cf. chromium oxide (Cr_2O_3) to Cr as shown in **Fig. S2**, where PBR = 2). However, when the PBR is close to unity, the formed metal layer becomes less porous leading to a relatively low electrochemical reduction speed (cf. TiO_2

to titanium in **Fig. S2**, where PBR = 1.33 based on TiO to titanium). When the PBR is much less than unity, deoxidation process will stall (cf. MgO to Mg in **Fig. S2**, where PBR = 0.81). It should be mentioned here, the final step in the process of electrochemical reduction of TiO_2 is TiO to titanium (as described in **RS7**), and thereby PBR for TiO to titanium is used to evaluate the kinetic difficulty level presented in the process.

Table S2. A comparison of different optimisation methods developed for the FFC-Cambridge process for titanium extraction [18-21]

Optimisation methods	Oxygen content in titanium (ppm by mass)	Energy consumption (kWh/kg Ti)	Current efficiency
Electrolyte composition ($CaCl_2$ + 2 mol% CaO), optimisation of polarisation regime (using a carbon pseudo-reference electrode to manipulate cathodic potentials), porous TiO_2 precursor (25 to 30% porosity), 16 hours of electrolysis [19].	3600	N/A	32%
	2400	N/A	15%
Ex situ perovskitisation, porous $CaTiO_3$ (40 to 50% porosity), 10 hours of electrolysis [20].	2100	N/A	28%
Highly porous TiO_2 precursor (68% porosity) achieved by using recyclable fugitive pore forming agent (NH_4HCO_3), and optimised dual-stage for electrolysis (3.2 V, 3 hours + 2.6 V, 3 hours at 850 °C) [18].	1900	21.5	32.3%
Inert anode, 14 to 16 hours of electrolysis, and optimised electrolysis conditions [21].	N/A	17	40%

Chloride process (using purer ore or rutile as the feedstock):

$TiO_2 + C + 2Cl_2 = TiCl_4 + CO_2$ RS8a

$TiO_2 + 2C + 2Cl_2 = TiCl_4 + 2CO$ RS8b

$TiCl_4 + O_2 = TiO_2 + 2Cl_2$ RS9

Sulphate process (using ilmenite, i.e. $FeTiO_3$, as the feedstock):

$FeTiO_3 + 2H_2SO_4 = FeSO_4 + TiOSO_4 + 2H_2O$ RS10

$TiOSO_4 + (n+1)H_2O = TiO_2{\cdot}nH_2O + H_2SO_4$ RS11

$TiO_2{\cdot}nH_2O = TiO_2 + nH_2O$ RS12

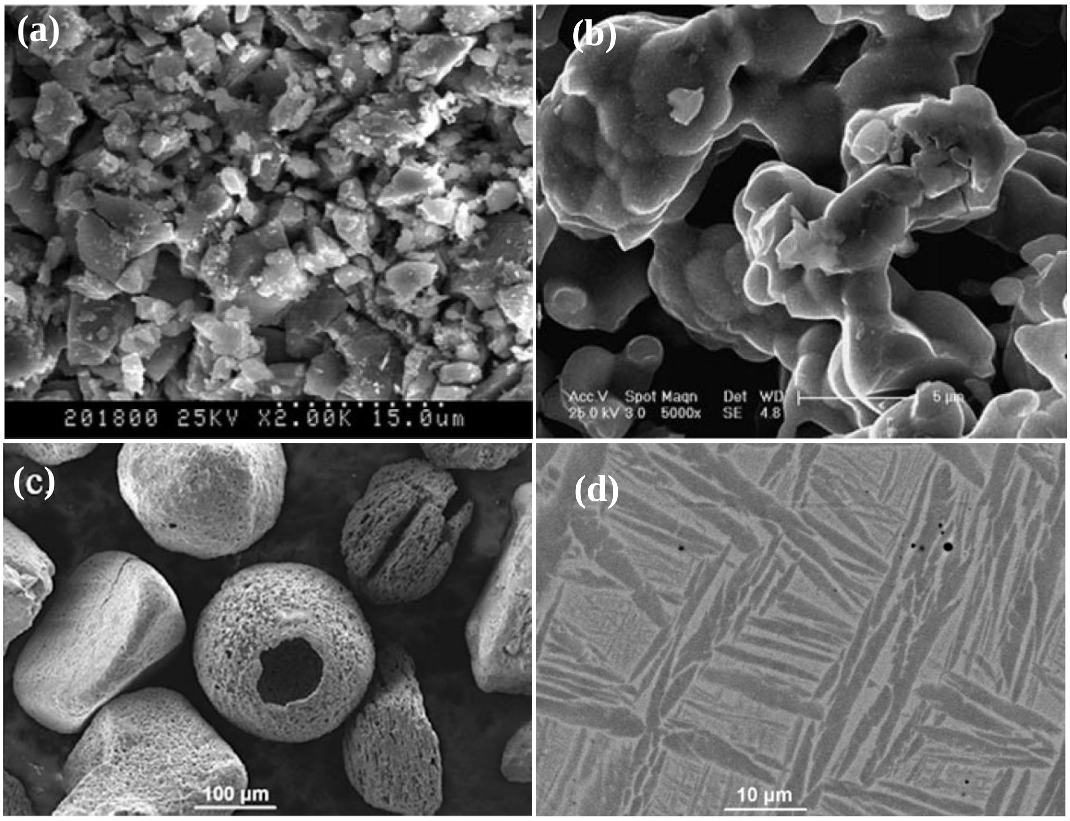

Fig. S3. Scanning electron microscopic images of (a) ground natural ilmenite feedstock, (b) its electrolysis product (electrolysis at 3.0 V, 900 °C, for 12 hours) (reprinted from [22] with permission), (c) synthetic rutile feedstock, and (d) backscattered image of consolidated (via spark plasma sintering) product from electro-reduction of synthetic rutile (with oxygen content ca. 3500 ppm) (reprinted from [23] with permission).

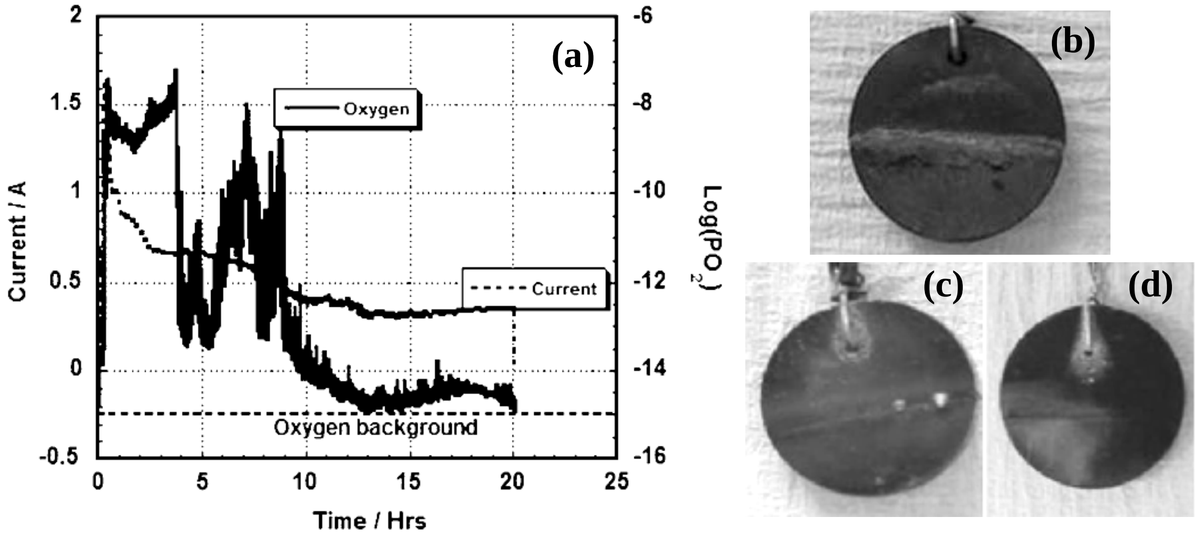

Fig. S4. (a) The current/oxygen-time plots for electrolysis of TiO_2 using a $CaRuO_3$ anode. Photos of (b) a pure $CaRuO_3$ anode after 150 hours electrolysis and washing, and (c, d) $CaTi_xRu_{1-x}O_3$ based inert anodes after electrolysis: (c) x = 0.72 and (d) x = 0.81 (reproduced from [24] with permission).

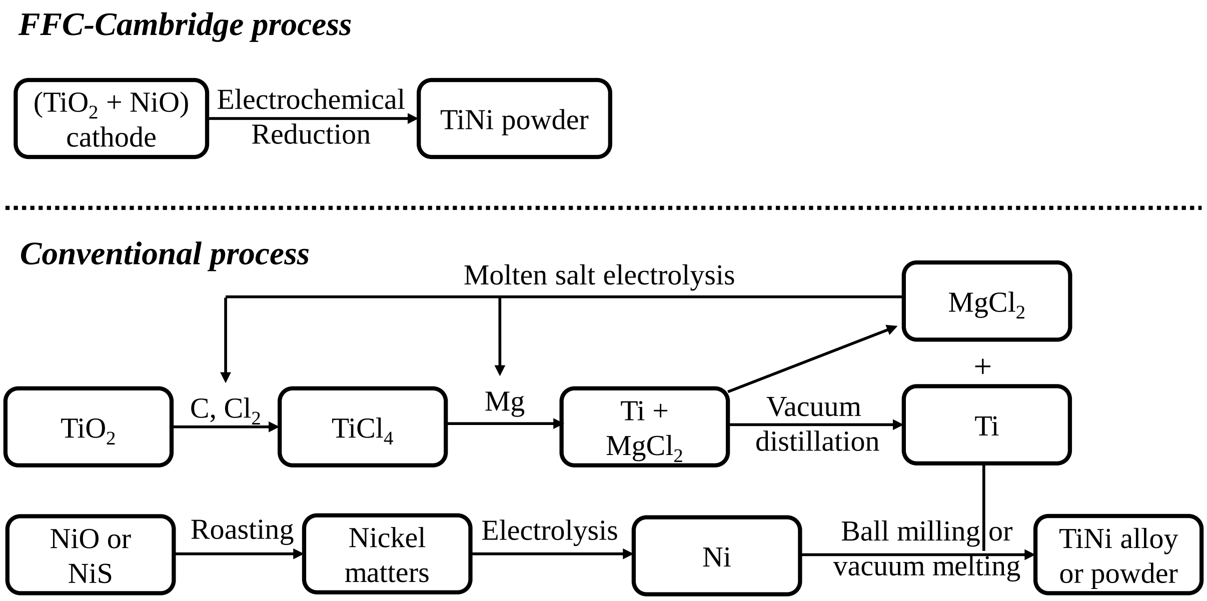

Fig. S5. Ti-Ni alloy production by the FFC-Cambridge process and a conventional process (reproduced from [25] with permission).

Commercial Progresses

Since 2001, Metalysis™ has been focusing on the commercialisation of the FFC-Cambridge process for titanium and other metals/alloys production. The titanium production capacity has been steadily increased from grams (GEN 1, R&D proof of concept, 2001 - 2003) to kilograms (GEN 2, Technical demonstrator, 2003 - 2009), then to tonnes (GEN 3, Commercial demonstrator, 2010 - 2014), and currently to 10s tonnes (GEN 4, Industrial scale production, 2015 - 2017) [26]. The GEN 5 facility will be the multiple modules of GEN 4, whose production capability can be easily adjusted from 100s to 1,000s tonnes according to different demands [26].

On the other hand, the FFC-Cambridge process has also been commercialised for production of silicon nanowire based negative electrode materials for Lithium-ion batteries by GLABAT™ (a Chinese state-owned automotive battery research institute) [27].

References

1. F. H. Froes and M. Ashraf Imam, *Key Eng. Mater.*, 436, 1 (2010).
2. D. Hu and G. Z. Chen, In *Advanced Extractive Electrometallurgy*, ed. C. Breitkopf and Swider-Lyons K. (Berlin, Heidelberg: Springer Berlin Heidelberg, 2017), p. 801.
3. D. J. Fray and C. Schwandt, *Mater. Trans.*, 58, 306 (2017).
4. R. B. Subramanyam, *Bull. Mater. Sci.*, 16, 433 (1993).
5. T. H. Okabe, T. Oda and Y. Mitsuda, *J. Alloys Compd.*, 364, 156 (2004).
6. F. H. Froes and B. Trindade, *J. Mater. Process. Technol.*, 153-154, 472 (2004).
7. G. Crowley, *Adv. Mater. Processes*, 161, 25 (2003).
8. I. Park, T. Abiko and T. H. Okabe, *J. Phys. Chem. Solids*, 66, 410 (2005).
9. J. C. Withers, J Laughlin and R. O. Loufty, *Light Metals 2007*, 1, 117 (2007).
10. R. O. Suzuki, K. Ono and K. Teranuma, *Metall. Mater. Trans. B*, 34, 287 (2003).

11. G. Z. Chen, D. J. Fray and T. W. Farthing, *Nature (London, U. K.)*, 407, 361 (2000).
12. I. Mellor, L. Grainger, K. Rao, J. Deane, M. Conti, G. R. Doughty and D. Vaughan, In *4 - Titanium powder production via the Metalysis process*, ed. F. H. Froes (Boston: Butterworth-Heinemann, 2015), p. 51.
13. U. Pal and A. Powell, *JOM*, 59, 44 (2007).
14. X. L. Zou, S. S. Li, X. G. Lu, Q. Xu, C. Y. Chen, S. Q. Guo and Z. F. Zhou, *Mater. Trans.*, 58, 331 (2017).
15. D. Wang, A. J. Gmitter and D. R. Sadoway, *J. Electrochem. Soc.*, 158, E51 (2011).
16. A. Allanore, L. Yin and D. R. Sadoway, *Nature (London, U. K.)*, 497, 353 (2013).
17. C. Schwandt and D. J. Fray, *Electrochim. Acta*, 51, 66 (2005).
18. W. Li, X. Jin, F. Huang and G. Z. Chen, *Angew. Chem., Int. Ed.*, 49, 3203 (2010).
19. C. Schwandt, D. T. L. Alexander and D. J. Fray, *Electrochim. Acta*, 54, 3819 (2009).
20. K. Jiang, X. Hu, M. Ma, D. Wang, G. QIU, X. Jin and G. Z. Chen, *Angew. Chem., Int. Ed.*, 45, 428 (2006).
21. C. Schwandt, G. R. Doughty and D. J. Fray, *Key Eng. Mater.*, 436, 13 (2010).
22. M. Ma, D. Wang, X. Hu, X. Jin and G. Z. Chen, *Chem. - Eur. J.*, 12, 5075 (2006).
23. L. L. Benson, I. Mellor and M. Jackson, *J. Mater. Sci.*, 51, 4250 (2016).
24. S. Jiao and D. J. Fray, *Metall. Mater. Trans. B*, 41, 74 (2010).
25. Y. Zhu, M. Ma, D. Wang, K. Jiang, X. Hu, X. Jin and G. Z. Chen, *Chin. Sci. Bull.*, 51, 2535 (2006).
26. Metalysis, “Technology”, http://www.metalysis.com/our-business/#gen5. Accessed 11 October 2017.
27. GLABAT, “Development of negative electrode materials”, http://www.glabat.com/article/content/view?id=23. Accessed 11 October 2017.
